# Comparative analysis of HPV16 gene expression profiles in cervical and in oropharyngeal squamous cell carcinoma

**DOI:** 10.18632/oncotarget.15977

**Published:** 2017-03-07

**Authors:** Andrea Cerasuolo, Clorinda Annunziata, Marianna Tortora, Noemy Starita, Giovanni Stellato, Stefano Greggi, Maria Grazia Maglione, Franco Ionna, Simona Losito, Gerardo Botti, Luigi Buonaguro, Franco M. Buonaguro, Maria Lina Tornesello

**Affiliations:** ^1^ Molecular Biology and Viral Oncology Unit, Istituto Nazionale Tumori IRCCS “Fondazione G. Pascale”, Napoli, Italy; ^2^ Gynecology Oncology Division, Istituto Nazionale Tumori IRCCS “Fondazione G. Pascale”, Napoli, Italy; ^3^ Department of Maxillofacial and Ear Nose and Throat Surgery, Istituto Nazionale Tumori IRCCS “Fondazione G. Pascale”, Napoli, Italy; ^4^ Department of Pathology, Istituto Nazionale Tumori IRCCS “Fondazione G. Pascale”, Napoli, Italy

**Keywords:** HPV16, E6 gene, E7 gene, cervical carcinoma, cervical intraepithelial neoplasia, Pathology Section

## Abstract

Human papillomavirus type 16 (HPV16) is the major cause of cervical cancer and of a fraction of oropharyngeal carcinoma. Few studies compared the viral expression profiles in the two types of tumor. We analyzed HPV genotypes and viral load as well as early (E2/E4, E5, E6, E6*I, E6*II, E7) and late (L1 and L2) gene expression of HPV16 in cervical and oropharyngeal cancer biopsies. The study included 28 cervical squamous cell carcinoma (SCC) and ten oropharyngeal SCC, along with pair-matched non-tumor tissues, as well as four oropharynx dysplastic tissues and 112 cervical intraepithelial neoplasia biopsies. Viral load was found higher in cervical SCC (<1 to 694 copies/cell) and CIN (<1 to 43 copies/cell) compared to oropharyngeal SCC (<1 to 4 copies/cell). HPV16 E2/E4 and E5 as well as L1 and L2 mRNA levels were low in cervical SCC and CIN and undetectable in oropharynx cases. The HPV16 E6 and E7 mRNAs were consistently high in cervical SCC and low in oropharyngeal SCC. The analysis of HPV16 E6 mRNA expression pattern showed statistically significant higher levels of E6*I versus E6*II isoform in cervical SCC (*p* = 0.002) and a slightly higher expression of E6*I versus E6*II in oropharyngeal cases. In conclusion, the HPV16 E5, E6, E6*I, E6*II and E7 mRNA levels were more abundant in cervical SCC compared to oropharyngeal SCC suggesting different carcinogenic mechanisms in the two types of HPV-related cancers.

## INTRODUCTION

Cancers of the cervix and of the head and neck region are among the most common tumors in the world accounting for approximately 528,000 and 686,000 new cases in 2012, respectively [1]. Although cervical cancer incidence has decreased over the last decades in many high-resource countries, due to the introduction of cervical screening programs, a stable or increasing incidence has been reported in low-income countries [2–4]. In parallel, the incidence of head and neck tumors, including larynx, hypopharynx and oral cavity carcinoma, has decreased as a consequence of smoking and alcohol use decline [5, 6]. However, during the same time period an increased incidence of oropharyngeal cancer has been observed in European countries, in Australia, Canada, and in the United States suggesting a new and emerging risk factor particularly among young men [7, 8].

Twelve high risk human papillomaviruses (HPV) have been recognized as the necessary cause of cervical cancer and of a subgroup of head and neck tumors. Indeed, the viral DNA has been identified in more than 99% of cervical carcinoma and in approximately 30% of head and neck tumors with a greater prevalence in the oropharyngeal SCC (45.8%) [9–12]. The HPV16 is the predominant cause of infection in both cervical and oropharyngeal SCC accounting for 65-70% and 82-87% of all HPV-positive cases, respectively, across the world [12–14].

The pathogenesis of oncogenic HPVs has been well studied in cervical neoplasia. The HPV infection initiates at the basal layer of the epithelium with the expression of early proteins E5, E6 and E7 which stimulate cell growth and viral DNA replication [15–19]. During the productive phase of HPV infection the viral genomes markedly replicate in the spinous layers and are encapsidated in the upper terminally differentiated epithelia where the late viral genes L1 and L2 are highly expressed [15, 20]. In cervical intraepithelial neoplasia grade 3 (CIN 3) and cervical carcinoma the virus undergoes abortive infection in which early genes E6 and E7 are expressed in all epithelial layers and cause the alteration of a number of cellular pathways involved in cell cycle regulation and apoptosis [15, 20, 21]. Specifically, the E6 protein binds to p53 oncosuppressor causing its ubiquitination and proteasomal degradation [22–24], and E7 oncoprotein abrogates pRB activity [25–27]. The HPV16 E5 protein induces dimerization of the epidermal growth factor receptor (EGFR) on the cell membrane concurring to the activation of mitogenic signals [18, 28].

The abortive infections are often accompanied by the integration of viral genome into human chromosomes determining the over expression of E6 and E7 oncoproteins either by the interruption of E2-mediated transcriptional repression or by functional alterations in the long control region [29–32].

Transcription of HPV16 E6 and E7 as well as E1, E2, E4 and E5 early viral genes, driven by enhancer elements located in the long control region and early promoter P97, produces multiple polyadenylated mRNAs containing exonic and intronic sequences which are alternatively spliced [33]. The alternative splicing of full-length polycistronic E6/E7 mRNAs, through the differential use of the splice donor at nucleotide 226 and splice acceptors at nucleotides 409 and 526, produces the E6*I and E6*II isoforms, respectively [33–35]. The E6*I and E6*II transcripts have shown to be more abundant in high grade neoplasia and invasive cancers than low grade cervical lesions [36]. The post-transcription regulation of HPV16 genes is controlled by several cellular splicing factors, including serine–arginine-rich (SR) proteins, heterogeneous nuclear ribonucleoproteins (hnRNPs), cleavage stimulation factor 64 kDa subunit (CSTF64) and CUG triplet repeat RNA-binding protein1 (CUGBP1), which are highly expressed in basal and middle layers cells of the cervical epithelium [37, 38]. No study has compared the HPV16 gene expression profiles in cervical and oropharyngeal SCC.

The aim of our study was to analyze the viral load and expression levels of HPV16 early genes, particularly E5, E6, E6*I, E6*II and E7 genes as well as L1 and L2 late genes in cervical carcinoma and in oropharyngeal cancer. We sought to identify similarities and differences in viral related transformation mechanisms between the two types of tumor.

## RESULTS

### HPV genotype distribution and viral load analysis

The study included women diagnosed with cervical SCC (n=28), borderline to mild dyscaryosis cytology (BMD) (n=40), CIN1 (n=66), CIN2 (n=4) and CIN3 (n=2) with a mean age of 52.6 (± 12.7), 40.2 (± 10.9), 38.2 (± 10.2), 32.8 (± 5.3), 22.5 (± 4.9) years at diagnosis, respectively. Six men (mean age of 52.3 [± 15.2] years) and four women (mean age of 58.5 [± 15.9] years) affected by oropharyngeal SCC, as well as three men (mean age of 45.7 [± 11.7] years) and one woman (41 years) with oropharyngeal dysplasia, were also included in the study. HPV DNA sequences were identified in 92.8 % (26 out of 28) of cervical SCC, 70% (7 out of 10) of oropharyngeal SCC, 65% (26 out of 40) of BMD, 65.3% (47 out of 72) of CIN, and 25% (1 out of 4) of oropharyngeal dysplastic tissues. Among the HPV positive samples the type 16 was the most prevalent representing 65.4% (17 out of 26) and 19% (9 out of 47) of all infections in cervical SCC and CIN, respectively, as well as 100% of all infections in oropharyngeal SCC and dysplastic tissues (Table 1). Other frequent genotypes were HPV33, 45, and 58 identified in 11.5% (3 out of 26), 7.7% (2 out of 26) and 7.7% (2 out of 26) of cervical SCC as well as HPV42 identified in 6.4% (3 out of 47) of CIN. The HPV sequences, detected in 57% (4 out of 7) and 44.4% (4 out of 9) of cervical and oropharyngeal pair matched non-tumor tissues, were always concordant with the genotype identified in the corresponding tumor. Samples positive for HPV16 were selected for further analyses.

**Table 1:** Clinic-pathological characteristics of patients with cervical and oropharyngeal SCC

Histopathology	HPV16	Other HPV genotypes	HPV negative
Cervical SCC^a^ (*n* = 28) Well differentiated (G1) (*n* = 1) Moderately differentiated (G2) (*n* = 9) Poorly differentiated (G3) (*n* = 16) Mean age (±SD) = 52.6 (± 12.7)	17 (60.7%) 1 (100%) 6 (66.7%) 8 (50%)	9 (32.1%) - 2 (22.2%) 7 (43.7%)	2 (7.1%) - 1 (11.1%) 1 (6.3%)
Cervical BMD^b^ (*n* = 40) Mean age (±SD) = 40.2 (± 10.9)	1 (2.5%)	25 (62.5%)	14 (35%)
CIN^c^1 (*n* = 66) Mean age (±SD) = 38.2 (± 10.2)	6 (9.1%)	36 (54.5%)	24 (36.4%)
CIN2 (*n* = 4) Mean age (±SD) = 32.8 (± 5.3)	2 (50%)	1 (25%)	1 (25%)
CIN3 (*n* = 2) Mean age (±SD) = 22.5 (± 4.9)	1 (50%)	1 (50%)	-
Oropharyngeal SCC (*n* = 10) Well differentiated (G1) (*n* = 1) Moderately differentiated (G2) (*n* = 1) Poorly differentiated (G3) (*n* = 5) Sex Men (*n* = 6) Women (*n* = 4) Mean age (±SD) Men = 52.3 (± 15.2) Women = 58.5 (± 15.9)	7 (70%) 1 (100%) 1 (100%) 2 (40%) 5 (83.3%) 2 (50%)	- - - - - -	3 (30%) - - 3 (60%) 1 (16.7%) 2 (50%)
Oropharyngeal dysplastic tissues (*n* = 4) Sex Men (*n* = 3) Women (*n* = 1) Mean age (±SD) Men = 45.7 (± 11.7) Women = 41	1 (25%) 1 (33.3%) -	- - -	3 (75%) 2 (66.7%) 1 (100%)

^a^squamous cervical carcinoma; ^b^borderline to mild dyscaryosis; ^c^cervical intraepithelial neoplasia.

**Figure 1:**
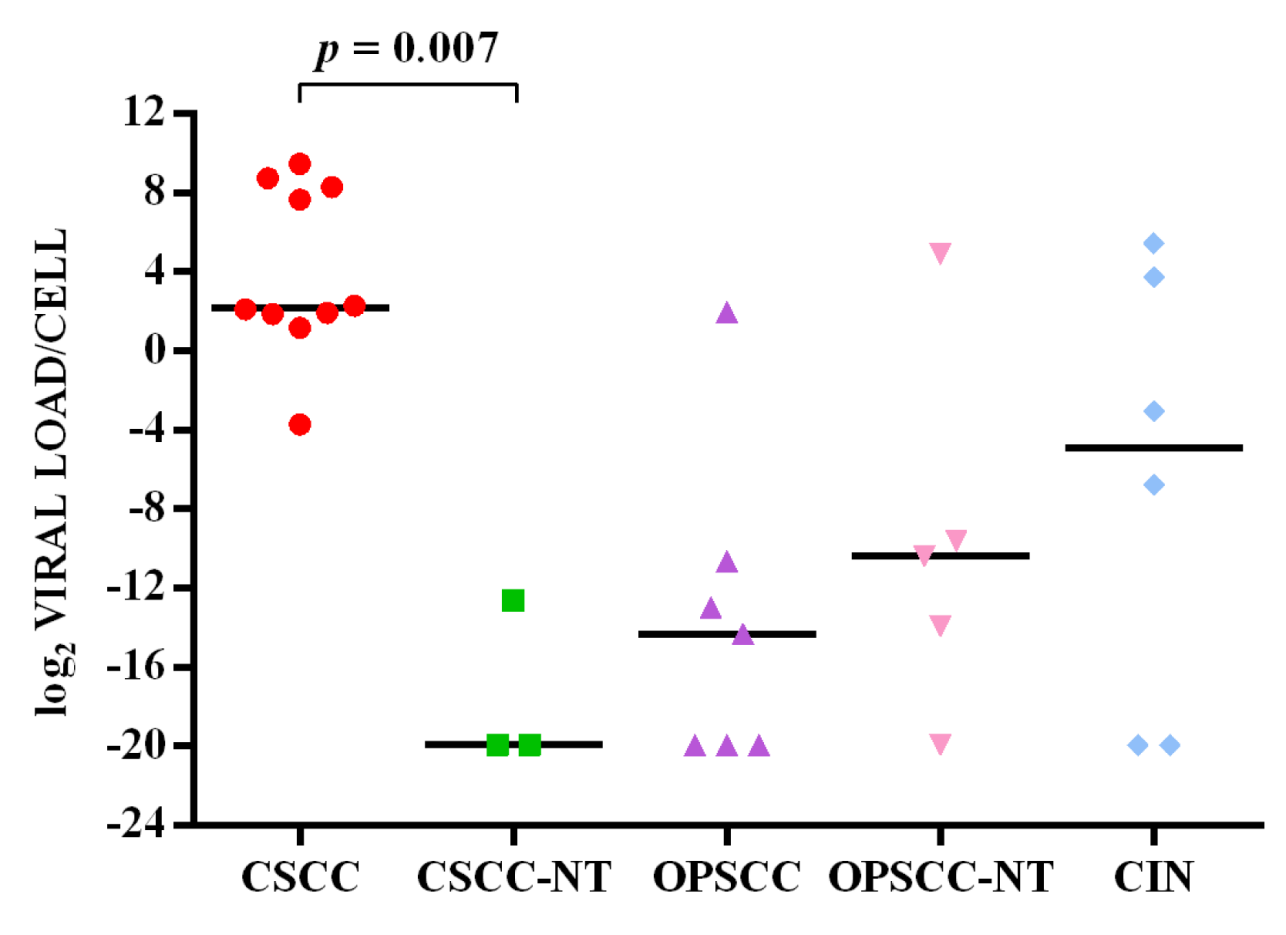
log^2^(HPV16 viral load/cell) in cervical SCC (CSCC), cervical paired non-tumor tissues (CSCC-NT), oropharyngeal SCC (OPSCC), oropharyngeal paired non-tumor tissues (OPSCC-NT) and cervical intraepithelial neoplasia (CIN) The horizontal line in the bars indicates the median.

HPV16 viral load ranged from <1 to 694 copies per cell in cervical SCC (median value 4.5 copies/cell) and from <1 to 43 copies per cell in CIN (median 0.1 copy/cell), while there was less than one copy per cell in paired non-tumor tissues (median 10^−6^ copy/cell). The viral load ranged from <1 to 4 copies per cell in oropharyngeal SCC (median 5×10^−5^ copy/cell) and from <1 to 30 (median 7×10^−4^ copy/cell) in paired non-tumor tissues. Less than one copy per cell was found in dysplasia and paired non-dysplastic tissues (Figure 1). The viral copy number was statistically significant higher in cervical SCC compared to paired non-tumor tissues (*p* = 0.007), but not compared to CIN. No significant differences were observed between viral load in oropharyngeal SCCs and respective paired non-tumor tissues (Figure 1).

### HPV16 gene expression analysis

**Figure 2:**
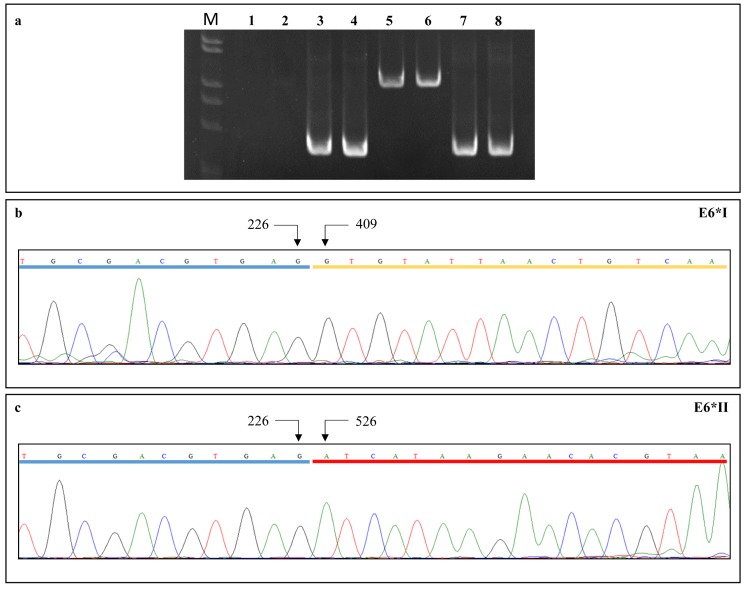
(a) PCR reactions using primer pairs targeting the full-length E6 cDNA (lanes 3-4) and genome viral DNA (lanes 7-8) PCR amplifications with primer pairs encompassing the untranslated region upstream the HPV16 P97 promoter gave negative results on cDNA (lanes 1-2) and positive results on viral DNA (lanes 5-6), suggesting the absence of DNA contamination in cDNA samples. (b) E6*I and (c) E6*II cDNA sequence electropherograms showing donor (nt 226) and acceptor (nt 409 for E6*I and nt 526 for E6*II) splicing nucleotides; exons are underlined.

Viral gene expression profiles were analyzed in all HPV16-positive samples by real time PCR using SiHa cell line cDNA as a positive control. The *GAPDH* cDNA was amplified in all samples to normalize the viral gene expression levels. The RNA quality was suitable for the analysis in 10 cervical SCC, six CIN, seven oropharyngeal SCC and one oropharyngeal dysplasia, along with three cervical and five oropharyngeal paired non-tumor tissues and one paired non-dysplastic tissue. Oligonucleotide pairs encompassing the untranslated region upstream the HPV16 promoter P97 were used to exclude the presence of HPV16 DNA in the cDNA samples (Figure 2).

**Figure 3:**
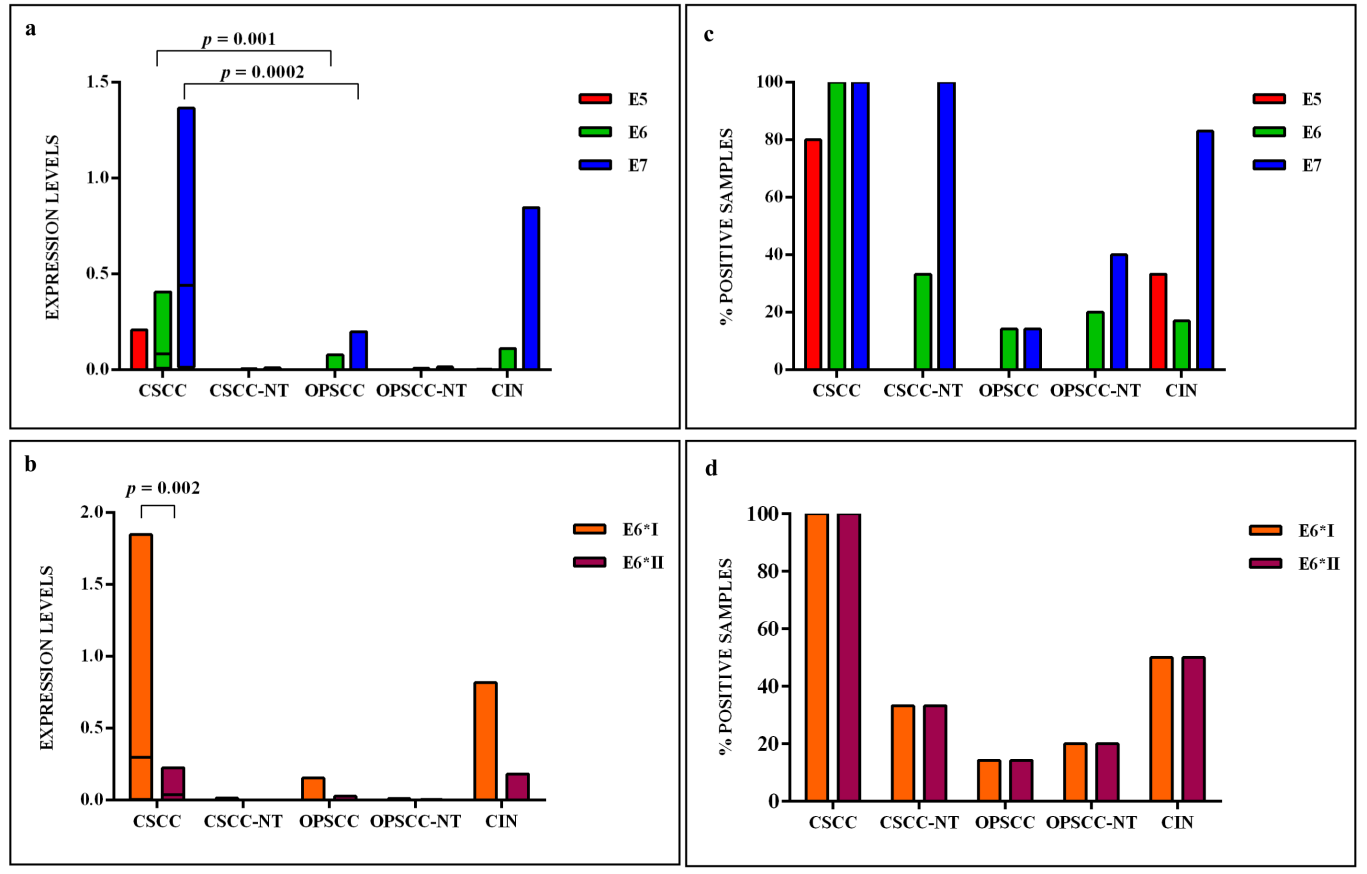
(a) The relative expression values, adjusted to GAPDH expression, of HPV16 E5, E6, E7 and (b) E6 isoforms, in cervical SCC (CSCC), paired cervical non-tumor tissues (CSCC-NT), oropharyngeal SCC (OPSCC), paired oropharyngeal non-tumor tissues (OPSCC-NT) and cervical intraepithelial neoplasia (CIN) The horizontal line in the bars indicates the median. (c) The percentage of cases expressing HPV16 E5, E6, E7 and (d) E6 isoforms in each histological group.

**Figure 4:**
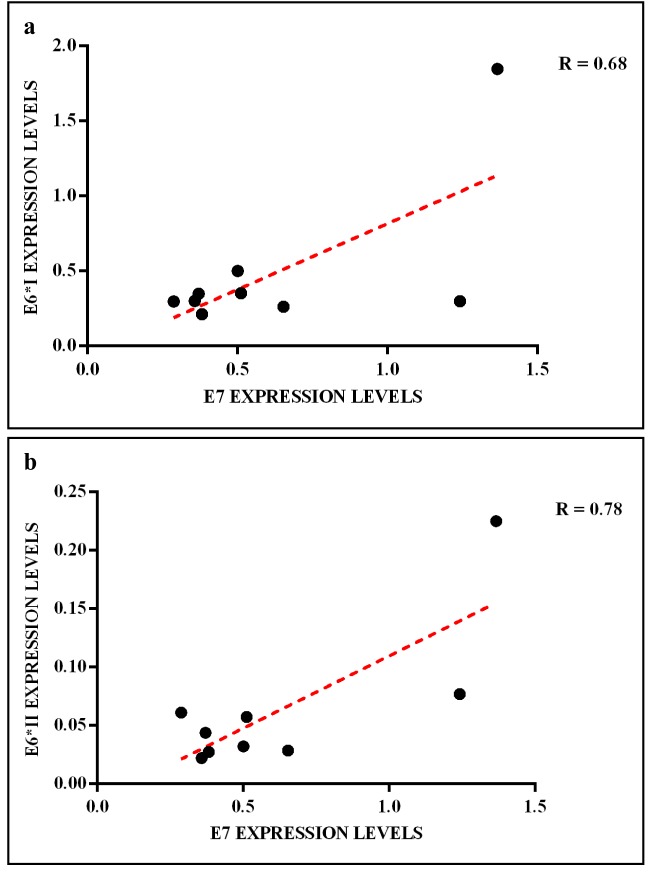
Linear correlation between (a) E6*I/E7 and (b) E6*II/E7 expression levels in cervical SCC

The HPV16 E6 full length mRNA was detected in all cervical SCC, in 14.3% (1 out of 7) of oropharyngeal SCC and 17% (1 out of 6) of CIN. The full E6 transcripts were also detected in 33.3% (1 out of 3) and in 20% (1 out of 5) of cervical and oropharyngeal paired non-tumor tissues, respectively (Figure 3). The E6*I and E6*II isoforms were both expressed in all cervical SCC, in 50% (3 out of 6) of CIN, in 14.3% (1 out of 7) of oropharyngeal SCC, in 33.3% (1 out of 3) and 20% (1 out of 5) of cervical and oropharyngeal paired non-tumor tissues, respectively (Figure 3). The analysis of E6 mRNA patterns showed that E6*I was more expressed than full-length E6 and E6*II in all samples, but the difference between E6*I and E6*II levels was statistically significant only in cervical SCC (*p* = 0.002). The oncogene E7 was highly expressed in all cervical SCC and paired non-tumor tissues, in 83% (5 out of 6) of CIN, in 14.3% (1 out of 7) of oropharyngeal SCC and in 40% (2 out of 5) of paired non-tumor tissues (Figure 3). The E6, E6*I, E6*II and E7 levels were significantly higher in cervical SCC than in paired non-tumor tissues, CIN and oropharyngeal SCC (*p* < 0.02). A positive linear correlation was observed in cervical SCC between E6*I and E7 expression levels (*R* = 0.68, *p* = 0.04) and between E6*II and E7 (*R* = 0.78, *p* = 0.01), (Figure 4). In oropharyngeal dysplasia only E6*I and E7 were found expressed, while none of viral mRNAs were detected in paired non-dysplastic tissues.

The HPV16 E2/E4 transcripts were detected at low levels in 80% (8 out of 10) of cervical SCC and in 17% (1 out of 6) of CIN, but not detected in oropharyngeal SCC. In one CIN only E4 mRNA and not E2 was revealed. The levels of E2/E4 and E5 mRNAs were higher in cervical SCC than in CIN, but the difference was statistical significant only for E2/E4 levels (*p* < 0.01). The E5 mRNA was identified in 80% (8 out of 10) of cervical SCC, in 33.3% (2 out of 6) of CIN, and in none of oropharyngeal SCC and paired non-tumor tissues (Figure 3).

L1 and L2 bicistronic transcripts were expressed at low levels in all cervical SCC and in 83% (5 out of 6) of CIN, however no expression was observed in oropharyngeal SCC and in paired non-tumor tissues.

There was no statistically significant correlation between viral load and oncogene expression in all analyzed samples.

## DISCUSSION

Persistent infection with high risk HPVs, mainly HPV16, is strictly associated with cervical SCC [39] and is becoming an emerging etiological factor for oropharyngeal SCC [40]. In this study the viral load and the expression pattern of HPV16 early (E2, E4, E5, E6, E6*I, E6*II, E7) and late (L1 and L2) genes were analyzed in cervical SCC and paired tissues, in CIN, in oropharyngeal SCC and paired non-tumor tissues, as well as in oropharyngeal dysplasia to uncover differences in HPV-related transformation mechanisms in different types of HPV-related tumors. In agreement with previous studies, we found that HPV16 was the most frequent genotype in cervical and oropharyngeal cancer [12, 14, 41].

Studies on the positive correlation between HPV16 viral load and cervical or oropharyngeal SCC are controversial [42–45], with some reporting that viral copy number increases with CIN severity and is highest in cervical SCC [46]. We found that HPV16 viral load was broadly variable especially in cervical SCC with a viral copy number significantly higher than in cervical paired non-tumor tissues. In oropharynx we found a slightly higher viral load in non-tumor tissues than in carcinoma. Until now no studies have compared the viral load in oropharyngeal SCC versus autologous non-tumor tissues. A recent study by Dang and Feng (2016) showed that oral and oropharyngeal cancer samples contained a higher HPV16 DNA load than normal tissues from non-cancer patients [47], however these data are not informative regarding the difference of viral copy number between non-tumor tissues and oropharyngeal carcinoma in the same patient.

Several studies showed that HPV gene expression pattern could be useful as viral molecular marker of tumor progression [48–50]. Analysis of E6 and E7 mRNA levels, together with other clinical data, seems useful to assess the risk of progression of cervical SCC and oropharyngeal SCC cases [43, 51, 52]. In our study, the E6 and E7 expression in oropharyngeal SCC and paired non-tumor tissues was much lower than in cervical SCC. These results are consistent with previous studies reporting that while HPV is always transcriptionally active in cervical SCC the viral oncogenes may not be expressed in HPV16 DNA positive oropharyngeal cancer suggesting that the virus in some HPV-positive head and neck cancers is not the main carcinogenic factor [53, 54]. Oropharyngeal SCC patients without biologically active HPV are considered at risk for cancer progression similarly to HPV-negative smoker patients [55–57].

Holzinger et al (2012) reported that both high viral load and increased expression of E6 and E7 mRNAs could define oropharyngeal SCC with active HPV16 involvement. However, we observed that there was no significant correlation between viral copy number and E6 and E7 viral mRNA levels in both cervical and oropharyngeal SCC [50]. Our observation is in agreement with previous studies reporting no correlation between DNA copy number and the E6 and E7 mRNA expression in cervical cancer, implying that not all viral genomes are transcriptionally active in tumors and cancer derived cell lines [58]. Paradigmatic is the highly divergent HPV16 copy number in CaSki (above 400 viral copies per cell) and SiHa cells (1 copy per cell) and the comparable levels of E7 transcripts (41.6 ± 1.5 and 14.8 ± 2.6 transcript copies per cell in CaSki and SiHa cells, respectively) in the two cervical cancer derived cell lines [59].

Recent in vitro molecular studies showed that E6* isoforms of high risk HPVs cooperate with E6 and are able to target the Discs Large MAGUK Scaffold Protein 1 (DLG1), the Pals1-Associated Tight Junction Protein (PATJ), the Membrane-Associated Guanylate Kinase Inverted 1 (MAGI-1), and to a lesser extent the Scribbled Planar Cell Polarity Protein (SCRIB), for proteasomal degradation, causing invasive growth of epithelial cells and disrupting epithelial cells junctions [60–62]. Several other E6 mRNA species, besides E6*I and E6*II, derived from alternative splicing have been identified, but their role in tumor progression has been poorly characterized [63–67].

The over expression of HPV16 E6*I and E6*II isoforms has been also shown to modify the levels of many proteins involved in mitochondrial dysfunction and oxidative phosphorylation in C33A cervical cells, and the β-integrin signaling pathway in HPV16-positive SiHa cells [66]. In high grade cervical intraepithelial neoplasia and in cervical cancer the E6*I is more expressed than E6*II [36, 68] and E6*I/E6*II ratio seems to be a predictive marker of clinical outcome in HPV-related oropharyngeal cancer [69].

In the present study the expression levels of HPV16 E6*I and E6*II were found high in all cervical SCC but not in oropharyngeal SCC. In agreement with other studies, we observed a linear correlation between E6*I and E7 mRNA as well as between E6*II and E7 mRNA [33, 70].

Interestingly, in the analyzed oropharyngeal dysplasia the E7 oncogene and the E6*I isoform were both detected leading to the hypothesis that HPV oncogenic activity may be important for the early phases of oropharyngeal neoplasia [53, 71]. It has been suggested that during the early stages of oropharyngeal carcinogenesis the viral oncoproteins may synergize with other carcinogens, such as smoke and alcohol, and may increase the risk of tumor progression [53, 71]. In our study the HPV16 E7 oncogene was much more expressed in cervical SCC than oropharyngeal SCC, suggesting that the expression of E7 is the main driver of tumor cell proliferation in cervical cancer but not in oropharyngeal SCC [72].

Several studies showed that E5 expression synergizes with E6 and E7 causing a more severe cancer phenotype [17, 73]. In our study the E5 expression was variable in cervical SCC, low or absent in CIN (except one sample showing also high E6 and E7 mRNA levels) and in cervical paired non-tumor tissues. Variable levels of E5 in cervical SCC could be due to the presence of both episomal and integrated forms of HPV16, as reported by Das et al. (2015) [74]. In fact, integration of HPV16 in the host genome could disrupt E5 ORF together with E2 ORF [75].

In oropharyngeal SCC and respective paired non-tumor tissues the E5 mRNA was found not expressed, in accordance to previous studies reporting variable expressions of E5 in head and neck cancer [73, 76]. The E2/E4 as well as L1 and L2 gene expression was generally low in cervical SCC and CIN and undetectable in oropharyngeal SCC [58, 77].

The main limitations of our study are the limited number of patients and the inability to evaluate long term disease outcome in relation to viral expression profiles. However, this is the first study comparing the HPV16 traits between the cervical and oropharyngeal SCC along with autologous non-tumor tissues.

In conclusion, this study confirmed that HPV16 is highly prevalent in cervical and oropharyngeal SCC. However the viral load is very low in oropharyngeal SCC compared to cervical SCC and importantly the viral oncogene mRNA levels and expression profiles are very different between cervical SCC and oropharyngeal SCC. Indeed, E5, E6, E6*I, E6*II and E7 mRNA were significantly more abundant in cervical SCC than in oropharyngeal SCC suggesting the presence of different carcinogenic mechanisms in the two different virus-related tumors.

## MATERIALS AND METHODS

### Patients and samples

Twenty-eight cervical SCC biopsies along with paired non-tumor tissues and 112 cervical biopsies, comprising 40 cases of borderline to mild dyscaryosis (BMD) cytology and normal histology, 66 cervical intraepithelial neoplasia (CIN) grade 1, four CIN2 and two CIN3 biopsies, obtained from patients attending the Gynecology Unit of Istituto Nazionale Tumori “Fond Pascale” from November 2013 to December 2015. Ten oropharyngeal SCC and paired non-tumor tissues, as well as four oropharyngeal dysplastic biopsies with paired non-dysplastic tissues, were obtained from patients referred to the Head and Neck Surgery Unit of the Istituto Nazionale Tumori “Fond Pascale” from January 2012 to December 2015 (Table 1). Each biopsy was divided in two sections: the first section was stored in RNA Later (Ambion, Austin, Texas) at -80°C, the second was subjected to histopathologic examination. Similarly, paired non-tumor biopsies were divided in two sections and processed for molecular analysis and histopathologic examination. This study was approved by the Institutional Scientific Board and by the Ethical Committee of the Istituto Nazionale Tumori “Fond Pascale”, and it is in accordance with the principles of the Declaration of Helsinki.

### DNA extraction, HPV genotyping and HPV16 viral load

Genomic DNA was extracted according to published procedures [78]. Specifically, 10 mg tissue samples were digested with proteinase K (150 μg per ml at 60°C for 30 min) in 100 μl of lysis buffer (10 mM Tris–HCl pH 7.6, 5 mM EDTA, 150 mM NaCl, 1% SDS), followed by DNA purification with phenol and phenol-chloroform-isoamyl alcohol (25:24:1) extraction and ethanol precipitation in 0.3 M sodium acetate (pH 4.6). The quantity of isolated DNA was spectrophotometrically assessed with Nanodrop 2000c (Thermo Fisher Scientific, Waltham, Massachusetts).

HPV detection was carried out by nested PCR amplifying 300 ng of genomic DNA with MY09/MY11 primer pairs [79] for the outer reaction and MGP primer system for the inner reaction in 50μl reaction mixture containing 5μl of outer reaction, as previously described [80]. HPV genotypes were identified by direct automated DNA sequencing analysis of MGP amplified products using the primer GP5+ [81] at Eurofins Laboratories (Milan, Italy). HPV type identification was performed by alignments of HPV sequences with those present in the GenBank database using the BLASTn software (http://www.ncbi.nlm.nih.gov/blast/html). HPV16 positive samples were selected for gene expression and viral load analysis.

HPV16 viral load quantization was performed in the Bio-Rad CFX96 Real-time PCR Detection System using 300 ng of template DNA, 12.5 µl of 1x iQ™ SYBR® Green supermix (Bio-Rad, Hercules, California) and 10 pmol each of E7 forward and reverse primers (Supplementary table) in a final volume of 25 µL. Thermal cycling consisted of a denaturation step at 95°C for 3 min, followed by 40 cycles of annealing at 54.3°C for 30 s, extension at 72°C for 30 s and denaturation at 95°C for 30 s. Exon 7 of *TP53* human gene was also amplified with primers targeting the exon 7 to normalize the viral load in each sample, as previously described [82]. Two replicates were performed for each sample and real-time PCR data were analyzed using Bio-Rad CFX manager software. Two standard curves were constructed to calculate absolute numbers of HPV16 E7 and *TP53* copies, respectively, by amplifying serial dilutions (10^5^ to 1 cell) of SiHa cell genomic DNA, containing 1 copy per cell of integrated HPV16 genome. The viral load per cell in each sample was calculated by normalizing the E7 copy number against the amount of cellular DNA (*TP53*) according to the formula: HPV copies/cell = Number of E7 copies/(number of *TP53* copies/2).

### HPV16 gene expression analysis

Total RNA was extracted from all samples using RNeasy MiniKit (Qiagen, Hilden, Germany) according to manufacturer procedure. The quality and quantity of isolated RNA was determined using the Nanodrop 2000c and calculating the ratio of absorbance at 260 nm and 280 nm. All RNA samples with a ratio in the range of 1.8-2.0 were included in further analyses. For each sample 250 ng of total RNA were reverse transcribed in 20 μL volume containing 4 μL of iScript reaction mix (Bio-Rad), 1 μL of iScript reverse transcriptase (Bio-Rad) and nuclease-free water. The reaction was incubated at 25°C for 5 min and at 42°C for 30 min, finally the enzyme was inactivated at 85°C for 5 min in the Gene Amp PCR System 2400 (Applied Biosystems, Foster City, California).

The cDNA samples were amplified for HPV16 early (E2/E4, E5, E6, E6*I, E6*II, E7) and late (L1 and L2) transcripts by real time PCR using specific primer pairs described in Supplementary table. The reverse primers specific to each E6 isoform encompassed the respective splicing acceptor nucleotides ensuring the specific amplification of each spliced isoform, as confirmed by nucleotide sequence analysis of PCR amplimers (Figure 2). The reaction mixture included 12.5 μL of 1x iQ™ SYBR® Green supermix (Bio-Rad), 10 pmol of each primer, 1 μL of cDNA and nuclease-free water in a final volume of 25 μL. All reactions were performed in duplicate. The amplifications were carried out on the Bio-Rad CFX96 real time PCR Detection System following the protocols described in Supplementary table. The expression of each viral gene was analyzed with the 2^−ΔCt^ method using *GAPDH* as a reference gene. The ΔCt values for each amplified transcript were calculated by subtracting the respective Ct value from the corresponding *GAPDH* Ct (ΔCt = Ct_x_ – Ct_GAPDH_). The Ct values were corrected for primer pairs efficiency to compare the expression levels of the different genes. Primer efficiency was calculated generating standard curves of SiHa cDNA serial dilutions for each analyzed gene.

### Statistical analysis

Statistical analysis was performed with GraphPad version 6 (Prism). Spearman's rank correlation coefficient (r) was calculated to evaluate correlation between viral load and oncogenes expression levels, Pearson's coefficient (R) was calculated to evaluate linear correlation between gene expression levels, while ANOVA Kruskal–Wallis test and U Mann–Whitney test were used to evaluate differences in gene expression levels and viral load. All variables with *p* < 0.05 were considered statistically significant.

## SUPPLEMENTARY MATERIALS FIGURES AND TABLES



## Abbreviations

HPV: Human Papillomavirus
SCC: squamous cell carcinoma
CIN: cervical intraepithelial neoplasia
