# Supplementary material for: Comparative analysis of HPV16 gene expression profiles in cervical and in oropharyngeal squamous cell carcinoma
Source: Oncotarget. 2017 Mar 7;8(21):34070–81. doi: 10.18632/oncotarget.15977 (PMC5470952; doi:10.18632/oncotarget.15977)
Supplement: Supplementary file 1 [file oncotarget-08-34070-s001.pdf]

Comparative analysis of HPV16 gene expression profiles in cervical and in oropharyngeal squamous cell carcinoma

Supplementary Material

Supplementary Table: PCR primers sequences used to amplify HPV16 cDNA ORFs, GAPDH cDNA and to exclude HPV16 genomic DNA contamination in cDNA samples.

Locus	Primers name	Sequences (5'-3')	Nucleotide Position (5')	Amplicon Size (bp)	Ta (°C)	References
ORFE2	HPV16E2F<br>HPV16E2R	AAGGACGGATTAACTGTAA<br>GTTGCCATTCACTATCATAT	3,581<br>3,781	201 bp	54.3	This study
ORFE4	HPV16E4F<br>HPV16E4R	CACACCACTAAGTTGTTG<br>TTACAGTTAATCCGTCCTT	3,508<br>3,599	92 bp	55.0	This study
ORFE5	HPV16E5F<br>HPV16E5R	GCATCCACAACATTACTG<br>TATCCACAATACTAATACCAATAG	3,867<br>4,000	135 bp	55.3	This study
ORFE6	HPV16RNAF1F2<br>HPV16RNAR1	AAGTTACCACAGTTATGC<br>TGTTCTAATGTTGTTCCAT	134<br>372	239 bp	54.5	This study
ORFE6*I	HPV16E6sI-IIFor<br>HPV16E6sIRev	AGGAGCGACCCAGAAAGTTA<br>GCTTTTGACAGTTAATACACCTCAC	120<br>428	107 bp	50.0	Pastuszak-Lewandoska, 2014
ORFE6*II	HPV16E6sI-IIFor<br>HPV16E6sIIRev	AGGAGCGACCCAGAAAGTTA<br>TACGTGTTCTTATGATCTCAGGTC	120<br>542	123 bp	50.0	Pastuszak-Lewandoska, 2014
ORFE7	HPV16E7F<br>HPV16E7R	ACAGAGCCCATTACAATA<br>CATTAACAGGTCTTCCAA	704<br>813	110 bp	54.3	This study
ORFL1	HPV16L1F<br>HPV16L1R	GCACGGATGAATATGTTG<br>CGCTGTGTATCTGGATTA	5,704<br>5,926	223 bp	55.3	This study
ORFL2	HPV16L2F<br>HPV16L2R	ATAGTCGCACAACACAAC<br>ATTATCCACATCTATACCTTCAT	4,917<br>5,026	92 bp	55.0	This study
HPV DNA (P97)	HPV16DNAF2<br>HPV16DNAR1R2	TAGTCATACATTGTTCAT<br>TTATCACATACAGCATAT	7,811<br>297	391 bp	54.5	This study
GAPDH	GAPDHs<br>GAPDHas	TTTAACTCTGGTAAAGTG<br>ATTTCCATTGATGACAAG	170<br>319	149 bp	45.0	This study

Nucleotide positions of primers used for HPV16 genes amplifications are given on the GenBank sequence number K02718.1. Nucleotide positions of primers used for GAPDH amplifications are given on the GenBank sequence number AF261085.1.
